# Supplementary material for: Genomic plasticity drives olfactory adaptation in a pest fly
Source: bioRxiv. 2025 Sep 29:2025.09.18.677102. Originally published 2025 Sep 19. Preprint. [Version 2] doi: 10.1101/2025.09.18.677102 (PMC12458127; doi:10.1101/2025.09.18.677102)

bioRxiv preprint doi: https://doi.org/10.1101/2025.09.18.677102; this version posted September 29, 2025. The copyright holder for this preprint (which was not certified by peer review) is the author/funder, who has granted bioRxiv a license to display the preprint in perpetuity. It is made available under aCC-BY 4.0 International license.

Supplementary Tables and Figures

Table 1. List of odorants

**Odorants**	**Source**	Catalog number
1-Heptanol	Millipore Sigma	72954
1-Nonanol	Millipore Sigma	74280
1-Octen-3-ol	Millipore Sigma	O5284
1-Propanol	Millipore Sigma	34871

bioRxiv preprint doi: https://doi.org/10.1101/2025.09.18.677102; this version posted September 29, 2025. The copyright holder for this preprint (which was not certified by peer review) is the author/funder, who has granted bioRxiv a license to display the preprint in perpetuity. It is made available under aCC-BY 4.0 International license.

2,3-Butanediol	Millipore Sigma	B84904
2-Methyl-1-butanol	Millipore Sigma	133051
2-Methyl-1-propanol	Millipore Sigma	538132
3-Methyl-1-butanol	Millipore Sigma	77664
2-Phenylethanol	Millipore Sigma	77861
6-Methyl-5-hepten-2-ol	Millipore Sigma	W488401
Ethanol	Millipore Sigma	459828
Farnesol	Millipore Sigma	43348
trans-2-Hexen-1-ol	Millipore Sigma	W256218
Butyraldehyde	Millipore Sigma	538191
Hexanal	Millipore Sigma	18109
Phenylacetaldehyde	Millipore Sigma	W287407
trans-2-Hexen-1-al	Millipore Sigma	132659
trans-2-nonenal	TCI America	N04835ML
trans,trans-2,4-nonadienal	TCI America	N05215ML
2,3-Butanedione	Millipore Sigma	11038
2-Acetoxy-3-butanone	Millipore Sigma	W352623
2-Heptanone	Millipore Sigma	537683
2-Nonanone	Millipore Sigma	W278505
3-Nonanone	Millipore Sigma	
3-Octanone	Millipore Sigma	136913
4-Methyl-3-hepten-2-one	Millipore Sigma	49722
Acetone	Millipore Sigma	90872
Acetophenone	Millipore Sigma	42163
2-Ethylhexanoic acid	Millipore Sigma	538701
2-Methylbutyric acid	Millipore Sigma	193070
2-Oxovaleric acid	Millipore Sigma	75950
3-Butenoic acid	Millipore Sigma	134716
Acetic acid	Millipore Sigma	A6283
Butyric acid	Millipore Sigma	B103500
Heptanoic acid	Millipore Sigma	W334804
Hexanoic acid	Millipore Sigma	153745
Isobutyric acid	Millipore Sigma	W222208
Linoleic acid	Millipore Sigma	62230
Myristic acid	Millipore Sigma	W276413
Octanoic acid	Millipore Sigma	C2875
Oleic acid	Millipore Sigma	O1008
Pentanoic acid	Millipore Sigma	75054
Propionic acid	Millipore Sigma	402907
trans-3-Hexenoic acid	Millipore Sigma	W317004
Benzyl acetate	Millipore Sigma	50475
Benzyl butyrate	Millipore Sigma	W214000
Butyl acetate	Millipore Sigma	W217417
Butyl butyrate	Millipore Sigma	W218618
Butyl hexanoate	Millipore Sigma	W220108

bioRxiv preprint doi: https://doi.org/10.1101/2025.09.18.677102; this version posted September 29, 2025. The copyright holder for this preprint (which was not certified by peer review) is the author/funder, who has granted bioRxiv a license to display the preprint in perpetuity. It is made available under aCC-BY 4.0 International license.

Butyl isovalerate	Millipore Sigma	W221805
cis-3-hexenyl acetate	Millipore Sigma	74597
cis-3-hexenyl butyrate	Millipore Sigma	W340210
cis-3-hexenyl lactate	Millipore Sigma	W369020
Citronellyl acetate	Millipore Sigma	W231118
Diethyl succinate	Millipore Sigma	W237701
Ethyl (methylthio)acetate	Millipore Sigma	W383503
Ethyl 3-(methylthio)propionate	Millipore Sigma	W334308
Ethyl 3-hydroxybutyrate	Thermo Scientific Chemicals	AC118540250
Ethyl 3-hydroxyhexanoate	Millipore Sigma	W354503
Ethyl acetate	Millipore Sigma	L000325ML
Ethyl benzoate	Millipore Sigma	W242217
Ethyl butyrate	Millipore Sigma	75563
Ethyl decanoate	Millipore Sigma	148970
Ethyl Heptanoate	Millipore Sigma	W243728
Ethyl hexanoate	Millipore Sigma	148962
Ethyl isovalerate	Millipore Sigma	71607
Ethyl lactate	TCI America	L000325ML
Ethyl octanoate	Millipore Sigma	W244910
Ethyl salicylate	Millipore Sigma	68291
Ethyl *trans*-2-butenoate	TCI America	C0418
Ethyl *trans*-2-octenoate	Millipore Sigma	W364304
Geranyl acetate	Millipore Sigma	173495
Hexyl-2-methylbutanoate	Millipore Sigma	W349909
Hexyl hexanoate	Millipore Sigma	W257206
Isoamyl acetate	Millipore Sigma	W205532
Isoamyl butyrate	Millipore Sigma	W206008
Isobutyl acetate	Thermo Scientific Chemicals	AAB2570009
Methyl benzoate	Millipore Sigma	18344
Methyl hexanoate	Millipore Sigma	259942
Methyl isobutyrate	Millipore Sigma	W269409
Methyl octanoate	Millipore Sigma	21719
Methyl salicylate	Millipore Sigma	76631
Pentyl acetate	Millipore Sigma	109584
Phenethyl propionate	Millipore Sigma	W286702
Phenyl acetate	Millipore Sigma	108723
trans-2-Hexenyl acetate	Millipore Sigma	W256404
α-Terpinyl acetate	Millipore Sigma	W304799
4-Ethylguaiacol	Millipore Sigma	39774
4-Methyl veratrole	Millipore Sigma	ALNH9A9DBBB4
Guaiacol	Millipore Sigma	W253200
Indole	TCI America	I002125G
p-Cresol	Millipore Sigma	42429
Veratrole	Millipore Sigma	W379905
Eugenol	Millipore Sigma	35995

bioRxiv preprint doi: https://doi.org/10.1101/2025.09.18.677102; this version posted September 29, 2025. The copyright holder for this preprint (which was not certified by peer review) is the author/funder, who has granted bioRxiv a license to display the preprint in perpetuity. It is made available under aCC-BY 4.0 International license.

Methyl Eugenol	Millipore Sigma	04607
1,4-Diaminobutane	Millipore Sigma	D13208
2-Phenylethylamine	Millipore Sigma	8073340005
Ammonia hydroxide	Millipore Sigma	338818
Cadaverine	Millipore Sigma	33211
Spermidine	Millipore Sigma	S2626
Pyridine	Millipore Sigma	27040
Pyrrolidine	Millipore Sigma	W352316
(+)-Limonene oxide	Millipore Sigma	218324
(R)-(+)-Limonene	Millipore Sigma	62118
(S)-(-)-Limonene	Millipore Sigma	62128
(-)-Menthone	Millipore Sigma	63677
(-)-*trans*-Caryophyllene	Millipore Sigma	75541
Farnesene	Millipore Sigma	W383902
Sabinene	Millipore Sigma	W530597
Terpinolene	Millipore Sigma	W304603
Valencene	Millipore Sigma	W344303
α-Ionone	Millipore Sigma	03642
α-Phellandrene	Millipore Sigma	W285611
α-Pinene	Millipore Sigma	147524
α-Terpineol	Millipore Sigma	04899
β-Citronellol	Millipore Sigma	C83201
β-Cyclocitral	Thermo Scientific Chemicals	AAB2570009
β-Ionone	Millipore Sigma	W259525
γ-Terpinene	Millipore Sigma	223190
γ-Decalactone	Millipore Sigma	93738
γ-Hexalactone	Millipore Sigma	68554
1-Heptadecene	Millipore Sigma	H1108
DEET	Millipore Sigma	36542
Diallyl disulfide	Millipore Sigma	30648

Table 2. List of primers for UAS constructs

	Forward primer (5' to 3')	Reverse primer (5' to 3')
DsuzOr22a	ATGTTAGCCAAATTTTTTCCGC	CTATTTAAACTTTTCGGCTAAG
DsuzOr23a-1	ATGAAACTCAGCAAGATTTCCG	TTATTTAAATGAAACTTGAGTTGGATG
DsuzOr23a-2	ATGAAACTCAGCAAGATCTTTG	TTACAAAAAAGAACCTTGGCCATC
DsuzOr23a-3	ATGGAACTCACCAAGATCCTTG	TTAAGTCCTCCGGTTTAGGGAG
DsuzOr23a-4	ATGGAACTCACCAAGATCCATG	TTATTTAAATGAAACTTGAGTTGGATG

bioRxiv preprint doi: https://doi.org/10.1101/2025.09.18.677102; this version posted September 29, 2025. The copyright holder for this preprint (which was not certified by peer review) is the author/funder, who has granted bioRxiv a license to display the preprint in perpetuity. It is made available under aCC-BY 4.0 International license.

DsuzOr23a-1+4	Or23a1 part: ATGAAACTCAGCAAGATTTCCG	Or23a1 part: tgttctttcactgactgagtttttgcagatggctggaaatgaaacttgagctggatggtc
	Or23a4 part: tcagtcagtgaaagaacaaaaccagacaggatggaactcaccaagatccttgagattgac	Or23a4 part: ttaaaatgaaactttagttggatg
DsuzOr67a-1	ATGATTGATTTAATAAAACAAAAAATTTTTG	TTACTTGTACATCATTCGAACGG
DsuzOr67a-2	ATGAGCATTGACTTAAAAAAGAAG	CTACTGATACATCATTCGAATGG
DsuzOr67a-3	ATGGCTATTAACTTGTTTAGAAAGG	TTACTGATACATTGTTCGAATGG
DsuzOr67a-4	ATGAGTGTCCAGTTTATCAAAAAAC	TTATTGACACACTGTTTTTATAGC
DsuzOr67a-5	ATGAGGGTCGACTCCTTGAAG	CTACTGATACATAGTTCGAAGAGC
DbiaOr67a-1	ATGGTTGTTGGCTTTCTAAAG	TTACTTATACATCATTCGAACAG
DbiaOr67a-2	ATGTGGATTAACTTCATAAGG	CTACTGATACATCATTCGAATG
DbiaOr67a-3	ATGGTCATTAAAGTATTTAGGAAG	TTACTGATACATCGTTCGAATG
DbiaOr67a-5	ATGAGTTTCGATTTCTTAAGG	TCACGGATACATAGTGCGAAG
Note: flanking regions were added to the forward (5'-CAACTTAAAAAAAAAAATCAAA-3') and reverse (5'-TAAAAACGATTCATTCTAGA-3') primers.		

Table 3. Primers for CRISPR

	Forward primer (5' to 3')	Reverse primer (5' to 3')
DsuzOr22a gRNA synthesize	TAATACGACTCACTATAGcactttcctctgaccaagca	TTCTAGCTCTAAAACtgcttggtcaggaaagtg
DsuzOr22a screen	tgccaatatcgattgtcgtg	atactgaccgtgggacaaaca
DsuzOr67a4 gRNA synthesize	TAATACGACTCACTATAGATCGATGCCATACGTTGAGT	TTCTAGCTCTAAAACACTCAACGTATGGCATCGAT
DsuzOr67a4 screen	CGAGTCCTCGAAACCTGAAG	ATGCCGAACACCACGTTTAT

bioRxiv preprint doi: https://doi.org/10.1101/2025.09.18.677102; this version posted September 29, 2025. The copyright holder for this preprint (which was not certified by peer review) is the author/funder, who has granted bioRxiv a license to display the preprint in perpetuity. It is made available under aCC-BY 4.0 International license.

Figure S1. Responses of ORNs housed in D. suzukii and *D. melanogaster* antennal Basiconic, and intermediate, and trichoid sensilla.
(A,B) Heatmaps of responses of ORNs housed in *D. suzukii* (A) and *D. melanogaster* (B) antennal basiconic, intermediate, and trichoid sensilla to 113 odorants. Each odorant was diluted in hexane and tested at a 10^{-2} dilution. Odorants are color coded by functional group.

Figure S2. Responses of 117 individual large basiconic sensilla in *DsuzOrco^3* to β-cyclocitral.

Figure S3. Generation of *DsuzOr67a4* mutant alleles.
(A,B) Schematics illustrating the generation of *DsuzOr67a4^1* (A) *and DsuzOr67a4^2* (B) mutants using CRISPR/Cas9-mediated gene disruption. Nucleotides in red represent the PAM site, underlined nucleotides indicate the gRNA sequence, and dashed lines denote deleted nucleotides.

(C) RNA sequencing data for *DsuzOr67a4* and *DsuzOr67a3*. Green boxes represent exons, and green lines represent introns.

Figure S1

bioRxiv preprint doi: https://doi.org/10.1101/2025.09.18.677102; this version posted September 29, 2025. The copyright holder for this preprint (which was not certified by peer review) is the author/funder, who has granted bioRxiv a license to display the preprint in perpetuity. It is made available under aCC-BY 4.0 International license.

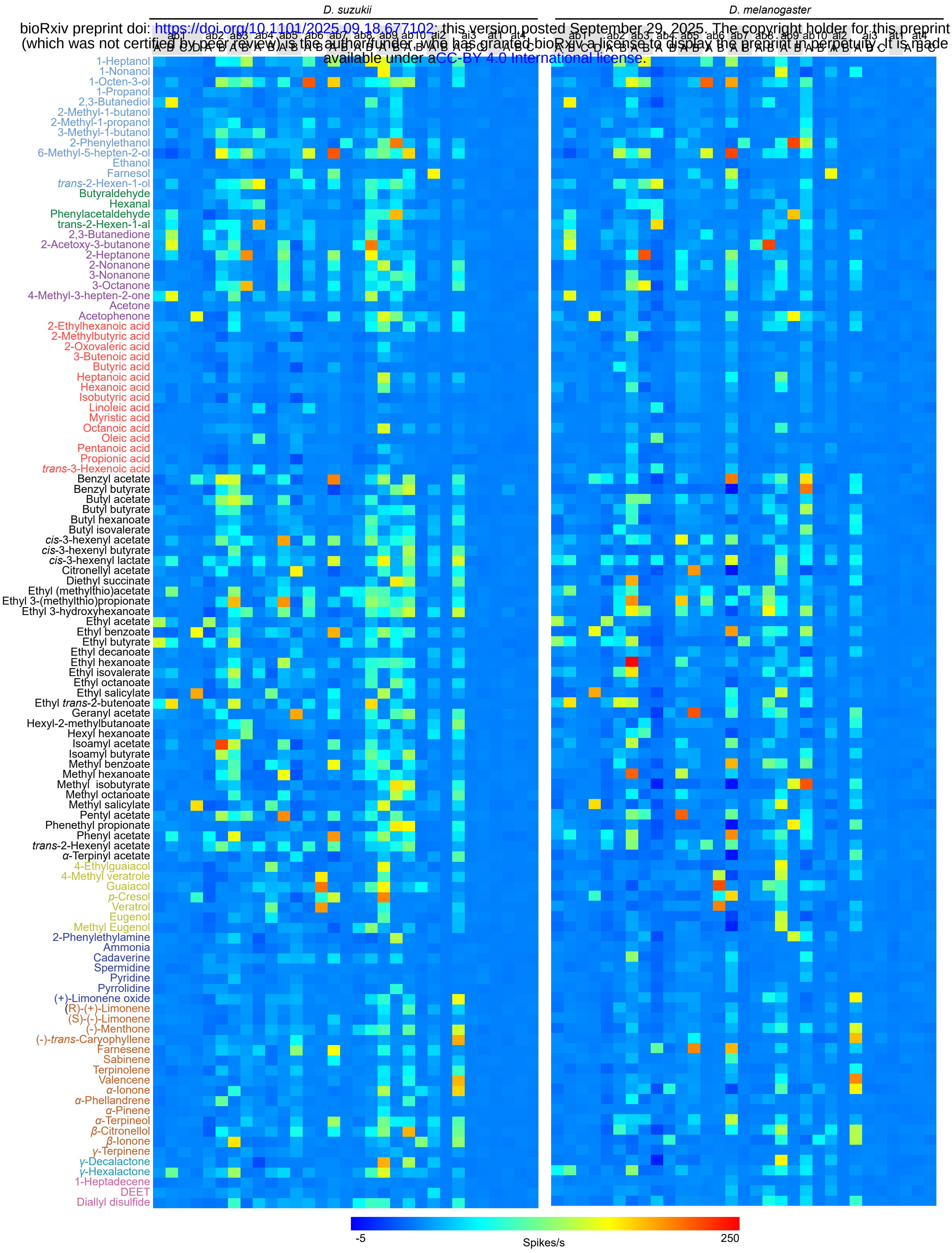

bioRxiv preprint doi: https://doi.org/10.1101/2025.09.18.677102; this version posted September 23, 2025. The copyright holder for this preprint (which was not certified by peer review) is the author/funder, who has granted bioRxiv a license to display the preprint in perpetuity. It is made available under aCC-BY 4.0 International license.

Figure S2

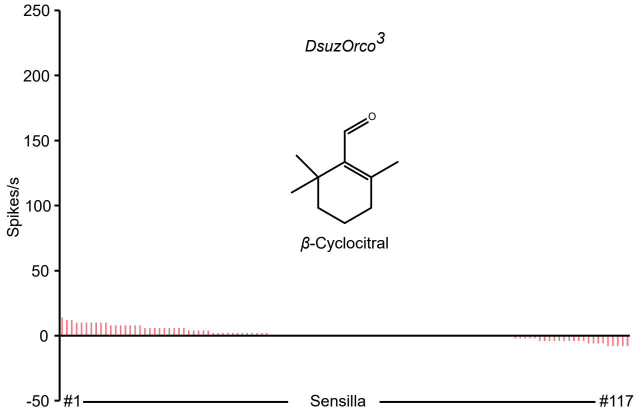

bioRxiv preprint doi: https://doi.org/10.1101/2025.09.18.677102; this version posted September 29, 2025. The copyright holder for this preprint (which was not certified by peer review) is the author/funder, who has granted bioRxiv a license to display the preprint in perpetuity. It is made available under aCC-BY 4.0 International license.

Figure S3

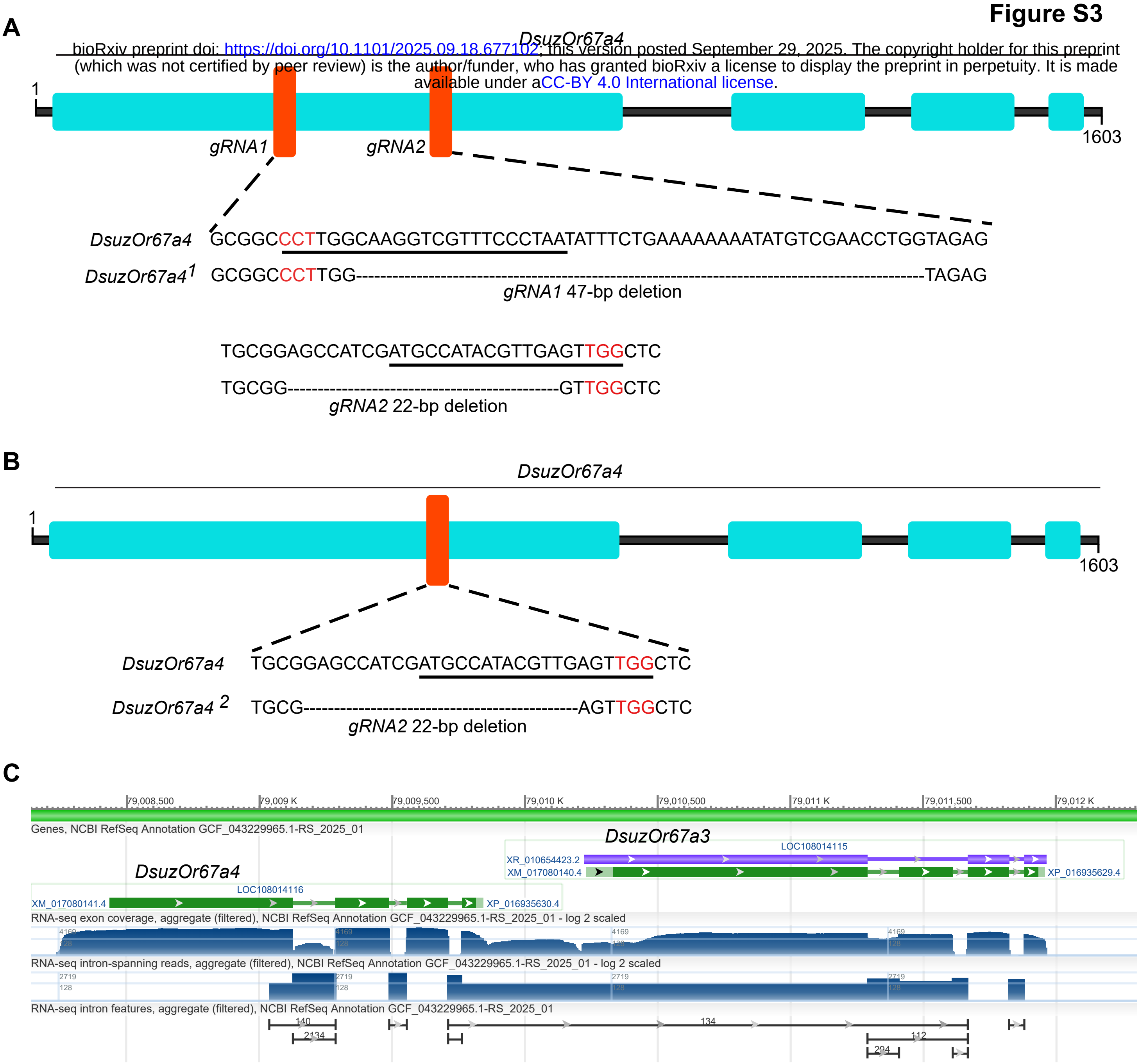

Supplement: 1 [file NIHPP2025.09.18.677102V2-supplement-1.pdf]
